# Early effects of the COVID-19 pandemic on physical activity and sedentary behavior in children living in the U.S.

**DOI:** 10.1186/s12889-020-09429-3

**Published:** 2020-09-04

**Authors:** Genevieve F. Dunton, Bridgette Do, Shirlene D. Wang

**Affiliations:** 1 Department of Preventive Medicine, Keck School of Medicine, University of Southern California, 2001 N. Soto St, Los Angeles, CA 90032 USA; 2 Department of Psychology, Dornsife College of Letters, Arts, and Sciences, University of Southern California, Los Angeles, USA

**Keywords:** Physical activity, Sedentary behavior, Children, Parents, Sex, Age, Locations, Online, Sports, COVID-19

## Abstract

**Background:**

COVID-19 restrictions such as the closure of schools and parks, and the cancellation of youth sports and activity classes around the United States may prevent children from achieving recommended levels of physical activity (PA). This study examined the effects of the COVID-19 pandemic on PA and sedentary behavior (SB) in U.S. children.

**Method:**

Parents and legal guardians of U.S. children (ages 5–13) were recruited through convenience sampling and completed an online survey between April 25–May 16, 2020. Measures included an assessment of their child’s previous day PA and SB by indicating time spent in 11 common types of PA and 12 common types of SB for children. Parents also reported perceived changes in levels of PA and SB between the pre-COVID-19 (February 2020) and early-COVID-19 (April–May 2020) periods. Additionally, parents reported locations (e.g., home/garage, parks/trails, gyms/fitness centers) where their children had performed PA and their children’s use of remote/streaming services for PA.

**Results:**

From parent reports, children (*N* = 211) (53% female, 13% Hispanic, M_age_ = 8.73 [*SD* = 2.58] years) represented 35 states and the District of Columbia. The most common physical activities during the early-COVID-19 period were free play/unstructured activity (e.g., running around, tag) (90% of children) and going for a walk (55% of children). Children engaged in about 90 min of school-related sitting and over 8 h of leisure-related sitting a day. Parents of older children (ages 9–13) vs. younger children (ages 5–8) perceived greater decreases in PA and greater increases in SB from the pre- to early-COVID-19 periods. Children were more likely to perform PA at home indoors or on neighborhood streets during the early- vs. pre-COVID-19 periods. About a third of children used remote/streaming services for activity classes and lessons during the early-COVID-19 period.

**Conclusion:**

Short-term changes in PA and SB in reaction to COVID-19 may become permanently entrenched, leading to increased risk of obesity, diabetes, and cardiovascular disease in children. Programmatic and policy strategies should be geared towards promoting PA and reducing SB over the next 12 months.

## Background

In March 2020, the respiratory disease caused by the SARS-Cov-2 virus, COVID-19, was declared a pandemic by the World Health Organization and a national emergency in the United States of America (U.S.). To date (Aug. 14, 2020), there had been 5.27 million COVID-19 cases and 167,000 related deaths recorded by the U.S. Centers for Disease Control and Prevention [1]. To prevent the spread of COVID-19, state and local governments enacted numerous restrictions on human movement and physical interactions. Starting mid-March, primary and secondary schools closed in all 50 states with many states extending school closures through the end of the 2019–2020 school year [2, 3]. As a result, children no longer had access to school-based physical activities such as physical education, recess, and walking to/from school. Youth team sports leagues cancelled all practices and games through May 2020 in most U.S. states with many states continuing these cancellations throughout the summer. Fitness and activity classes for youth such as gymnastics, dance, and martial arts were also cancelled or postponed through mid-May or later. Federal, state, and local public parks, playground, trails, and beaches were closed in many jurisdictions starting mid-to-late March with some re-openings occurring in late April through mid-May. Although these social-distancing measures were necessary to slow the spread of COVID-19, they may have limited children’s ability to engage in sufficient levels of physical activity (PA) to maintain health and prevent disease.

Promoting adequate levels of PA in children is a major public health issue. Recent estimates suggest approximately three-quarters of U.S. children and youth between the ages of 6 and 15 years fail to meet the 2018 Physical Activity Guidelines for Americans recommendation of at least 60 min of moderate-to-vigorous physical activity (MVPA) per day [4]. Additionally, nearly half of U.S. children and youth between the ages of 6 and 11 years engage in two or more hours of screen time per day–a level of behavior greater than recommended levels put forth by the American Academy of Pediatrics [5]. Insufficient PA and excessive sedentary behavior (SB) among children represents a significant problem because health behavior patterns in childhood are likely to persist into adulthood and can lead to increased risk for a number of serious health conditions (e.g., overweight/obesity, type II diabetes, and metabolic syndrome) in later childhood and adulthood [6].

It is unclear how COVID-19 related closures, cancellations, and restrictions have impacted PA participation among U.S. children. The cancellation of youth sports and activity classes have inspired programs, coaches, independent fitness professionals, and other entities to offer online streaming services with live or recorded sports/activity classes for youth using platforms such as Zoom, YouTube, Instagram, and proprietary mobile applications. Furthermore, without the structure of school or demands of after-school lessons and classes, some children may have more time for physically active free play at home. However, there may be enormous disparities in access to these opportunities based on household financial considerations, digital technology access, house and yard size, and neighborhood safety and traffic volume [7–11]. It has been argued COVID-19 school closures will lead to increased rates of obesity in children [12, 13] in part because schools provide opportunities and facilities for physical activity through physical education and recess [14–16]. Although policies vary, many states require between 90 and 150 min per week of physical activity during the school day [16, 17]. As a result of these policies, children typically engage in lower levels of PA and more sedentary time on weekend days as compared to school days [18, 19]. Also, children tend to gain more weight over the summer, especially children who are not enrolled in structured summer camps and activities [20–23]. If COVID-19 school closures and sport team/activity class cancellations lasting a year or more result in similar inactivity patterns that are typically seen on weekend days and during the summer, there may be enormous consequences for children’s overall physical health.

Therefore, the purpose of this study was to investigate the effects of the COVID-19 pandemic on PA and SB in U.S. children (ages 5–13 years) during the spring of 2020. The first objective was to examine differences in children’s PA and SB during the early-COVID-19 period (April–May 2020) by child sex and age. The second objective was to examine parent-reported changes in these behaviors from the pre-COVID-19 period (February 2020) to the early-COVID-19 period by child sex and age. The third objective was to investigate changes in the locations of children’s PA between the pre-COVID-19 period and the early-COVID-19 period by child sex and age. An ancillary goal was to examine rates of the use of remote and streaming services for PA by child sex and age. Given the potential for increased risk of obesity, diabetes, and other chronic diseases in children due to prolonged physical inactivity, information about the impact of the COVID-19 pandemic on children’s PA can inform immediate programmatic and policy efforts during the next few months of the pandemic.

## Methods

### Study design

A prospective survey design assessed the effects of the COVID-19 pandemic on PA among U.S. children by using online parent-reported surveys. A baseline online survey was completed between April 25 – May 16, 2020, and a second online survey is scheduled to occur within 6–12 months. The current analyses focus on data collected from the baseline online survey.

### Recruitment and participants

A convenience sampling strategy, focused on recruiting a general population of parents living in the U.S. during the COVID-19 pandemic, was utilized. To avoid in-person interactions, potential respondents were electronically invited through various social media platforms (e.g., Facebook, Twitter) and university-based email list servs of students, faculty, and staff. Inclusion criteria were as follows: 18 years or older, able to speak and read English, live in the U.S., is a parent or legal guardian of a child between the ages of 5–13, greater than or equal to 50% of child’s custody resides with the parent/legal guardian, and planned parental custody for the next 12 months. Individuals were directed to an online screening form directly from an email or social media post. Once eligibility was determined, individuals agreed to participate through an anonymous online information sheet describing the study procedures, risks, and benefits. The Institutional Review Board of the University of Southern California determined the study procedures presented no more than minimal risk and approved it as exempt from full review.

### Procedures

Participants completed the online screening form, information sheet, and baseline survey in English through an online survey platform, Research Electronic Data Capture, which conforms to HIPAA security requirements for the protection of data [24, 25]. Baseline data collection took place over 22 days (April 25 – May 16, 2020). The baseline survey took approximately 20 min to complete. Participants had the option to complete it either on their mobile phone, tablet, or desktop device. Each parent reported on one child of theirs who was between ages 5–13. If a parent had more than one child between ages 5–13, they were asked to only report on the one child whose birthday occurred next chronologically after the date the survey was administered. Upon completion of the baseline survey, participants were eligible to be entered into a lottery to win one of ten $50 gift cards.

### Measures

The current analyses utilized data on child’s past day PA and SB, parents’ perceived changes in level of PA and sitting time, locations of child’s PA, child’s use of remote/streaming services, and demographics.

### Children’s physical activity and sedentary behavior

Parents completed a measure of their child’s previous day PA created for the purposes of capturing non-school based activities frequently occurring during COVID-19, modeled upon the structure and format of previous day PA measures used in youth [26]. Parents also completed a measure of their child’s previous day SB with an instrument used in the “Active Where” survey [27]. The instructions asked parents to indicate how much time on the previous day their child performed each from a list of 11 types of common non-school-based PA and 12 common types of non-school-based SB for children. They were asked to think about the time that their child was awake on the previous day. The SB instrument has demonstrated acceptable intraclass correlations and test-retest reliability in previous research [27]. The specific types of PA included: sports practice or training; activity classes/lessons; free play or unstructured PA; jogging or running; biking; scootering, skateboarding, or roller skating; swimming; going for a walk; circuit training or conditioning; weightlifting, or other (write-in). Types of SB included: watching television, videos, or movies; playing computer or video games; using the internet, emailing, or other electronic media for leisure; doing school-related video calls; doing video calls; doing school-related work; sitting while listening to music; sitting while talking on the telephone or texting; sitting while hanging out or talking with friends or family in person; reading a book or magazine NOT for school; doing inactive hobbies; and riding or driving in a car. Duration in each activity was reported. After completing the measures, parents were asked to indicate how similar their child’s level of PA or sitting YESTERDAY, respectively, was compared to the past 7 days.

The Youth Compendium of Physical Activities (Youth Compendium) was utilized as a guide to calculate metabolic equivalents (METs) for each of the 11 types of PA. METy values for the age group 6–9 were applied for children who were 5 years old. If the type of PA in the baseline survey was not explicitly stated in the Youth Compendium, the METy values for the activity that most closely resembled the type of PA were used (e.g., the METy values for “jumping jacks” were utilized for the survey item “circuit training/conditioning). Five items on the survey were not explicitly stated in the Youth Compendium. If a participant wrote in an activity for ‘other’, METy values were inputted for each individual case. To calculate MET-minutes (MET-mins) for each of the types of PA, each age-group specific mean METy value was multiplied by the number of minutes parents reported their child performed in the previous day. Daily total MET-mins of PA were calculated for each child by taking the sum of MET-mins across the 11 specific types of PA.

Children’s previous day sitting/SB was examined by calculating the minutes spent in each of the 12 types of SB. Daily total minutes of SB were calculated for each child by taking the sum of minutes across the 12 types of SB. If the daily total minutes exceeded 1080 min, the value was truncated to 1080 min to address outliers. Daily total minutes of SB was also separated into two categories: minutes of school-related sitting (i.e., doing school-related video calls and doing school-related work) and minutes of sitting for leisure (i.e., all other types listed in the baseline survey).

### Perceived changes in children’s levels of physical activity and sedentary behavior

Parents were asked to compare their child’s current levels of PA (i.e., past 7 days) and SB to the pre-COVID-19 period (i.e., February 2020). Specifically, they were asked, “Compared to Feb 2020, how physically active has your child been/how much sitting has your child been doing in the PAST 7 DAYS?” Response options were reported on a 5-point likert scale ranging from a “much more physically active in past 7 days as compared to February 2020,” to “much less physically active in past 7 days as compared to February 2020.”

### Locations of children’s physical activity

Parents were asked to report the types of locations in which their child did PA in February 2020 and over the past 7 days (i.e., “where did your child do physical activity?”) with instructions to choose all that apply from the following options: inside my home or garage, in my yard or driveway, on the sidewalks and roads in my neighborhood, on the sidewalks and roads outside my neighborhood, gym or fitness center, at a park or trail, at an indoor sports facility (e.g., basketball/tennis court, ice rink), or at an outdoor sports facility (e.g., basketball/tennis court, baseball diamond). These settings were based upon locations assessed in the “Active Where” survey [28, 29] and correspond to where children frequently engage in physical activity based upon Global Positioning Systems (GPS) [30].

### Children’s use of remote/streaming services for physical activity

Parents reported whether their child used remote or streaming services to participate in PA during the during the early-COVID-19 period. Specifically, they were asked, on how many days of the past 7 days did their child participate in any team sports training sessions or practices, activity classes or lessons classes or sessions provided by a health club or gym through remote services, such as streaming classes via the internet or mobile applications.

### Demographics

Parents reported on their child’s biological sex at birth (male vs. female), birthdate, grade in school, ethnicity (Hispanic vs. non-Hispanic), and race (coded as American Indian or Alaska Native, Asian, Black, Native Hawaiian or Pacific Islander, White, Mixed race, Other). Parents also reported on their own birthdate, gender, marital status (coded as married vs. not married), employment status (coded as works full-time vs. does not work full-time), and annual household income (categorized as less than $24,999, $25,000–$54,999, $55,000–$94,999, $95,000 or more). All survey questions allowed participants to choose the option ‘do not know/prefer not to answer’.

### Statistical analyses

Prior to data analyses, variables were screened for violations of statistical assumptions (e.g., normality, linearity). Variables representing the duration of participation in specific types of physical activities were highly skewed due to the substantial number of children who did not perform any type of activity. Therefore, these variables were coded as some vs. none for subsequent analyses. The total MET-min PA variable, and all the SB variables (i.e., specific types, total sitting minutes, minutes of school-related sitting, minutes of sitting for leisure) were also positively skewed and thus subjected to square root transformations. To test the first objective, chi-square and independent samples t-tests compared rates and means for participation in the specific types of PA and SB during the early-COVID-19 period (April – May 2020), respectively, by child sex (male vs. female) and child age group (5–8 years vs. 9–13 years). Multiple linear regression analyses further examined whether child sex and age predicted total MET-min, total sitting minutes, minutes of school-related sitting, minutes of sitting for leisure) after controlling for child ethnicity (Hispanic vs. non-Hispanic), parent employment status (works full-time vs. does not work full-time), parent marital status (married vs. not married), and annual household income. To test the second objective, ordinal logistic regression models examined whether child sex and age predicted the likelihood of parents perceiving changes (i.e., much more, somewhat more, about the same, somewhat less, much less) in PA and SB between the pre-COVID-19 period (February 2020) and early-COVID-19 period (April–May 2020) after adjusting for the same demographic covariates listed in the first objective. The third objective was tested by using generalized estimating equations (GEE) to examine within-subject changes in the likelihood of children engaging in PA at various locations differed by child sex and age group. The within-subject factor was Time (i.e., pre-COVID-19 vs. early-COVID-19), the between-subject factors were child sex and age group, and covariates included child ethnicity (Hispanic vs. non-Hispanic), parent employment status (works full-time vs. does not work full-time), parent marital status (married vs. not married), and annual household income. Interactions were tested for Time × Child sex and Time × Child age group. To address the ancillary goal of examining whether the likelihood of children engaging in some vs. none for each of three types of PA remote and streaming services (i.e., team sports, activity classes and lessons, classes offered by gym) also differed by child sex and age group, logistic regressions were conducted controlling for the covariates used in the above models.

## Results

### Data availability and demographic characteristics

A total of 325 parents expressed interest in the study and completed the screening questions. Of this number, *n* = 41 individuals were not eligible. Twenty-seven individuals were no longer interested in the study after completing the online screening form, and 257 individuals agreed to participate in the study. Thirty-one cases were removed that had not yet completed the baseline survey at the time of data analysis, and 14 participants were missing data on child PA and SB, leaving an analytic sample size of 211. Additional participants were missing data on demographic variables, but they were retained in the analytic sample and treated with pairwise deletion. Table 1 shows the descriptive statistics for the demographic characteristics of the analytic sample. Individuals resided across 35 U.S. states and the District of Columbia. Parents ranged in age from 20 to 61 years, with an average age of 42.05 years (*SD* = 5.34). Children’s ages ranged from 5 to 13 years, with an average age of 8.71 years (*SD* = 2.58). Most parents who participated were mothers, identified as non-Hispanic, and had graduated from college. About half of the children reported on in the study were female and the majority were non-Hispanic. Over half of parents reported working full-time. Over half of the sample reported an annual household income of $95,000 or more.

**Table 1:** Descriptive statistics for sample on demographic characteristics

Variable	*n (%)*
Child age in years (Mean ± SD)	8.73 + 2.58
Child Sex	
Male	100 (47.39)
Female	111 (52.61)
Child Ethnicity^a^	
Hispanic	28 (13.27)
Non-Hispanic	181 (85.78)
Child Race^b^	
White	162 (76.78)
Black	3 (1.42)
Asian	8 (3.79)
American Indian or Alaskan Native	0 (0.00)
Native Hawaiian of Pacific Islander	1 (0.47)
Mixed race	31 (14.69)
Other	2 (0.95)
Parental Marital Status^c^	
Married	174 (82.46)
Not married	24 (11.37)
Parent Work Status^c^	
Works full-time	129 (61.14)
Does not work full-time	69 (32.70)
Annual Household Income^d^	
Less than $24,999	4 (1.90)
$25,000 - $54,999	9 (4.27)
$55,000 - $94,999	33 (15.64)
$95,000 or more	144 (68.25)

*N* = 211. ^a^Missing for 2 participants; ^b^Missing for 4 participants; ^c^Missing for 13 participants; ^d^Missing for 21 participants

### Children’s physical activity and sedentary behavior during the early-COVID-19 period

Table 2 shows descriptive statistics for the frequencies of children who performed various types of PA on the previous day during the early-COVID-19 period (April–May 2020). The most frequently reported physical activities were free play/unstructured PA (e.g., running around, tag, other active games) and going for a walk. Child sex and age differences were observed in the frequencies of reported physical activities (see Table 2). Boys were more likely to participate in sports practice/training than girls. Additionally, younger children (ages 5–8) were more likely to participate in free play/unstructured physical activity, biking, and scootering/skateboarding/roller skating than older children (ages 9–13). However, older children were more likely to participate in circuit training/conditioning than younger children. Descriptive statistics for the duration (in minutes) of various types of sedentary behaviors performed by children on the previous day during the early-COVID-19 period (April–May 2020) are shown in Table 3. Children spent the most time watching television/videos/ movies, sitting while hanging out with friends and family in person, doing school-related work, and playing computer or video games. Child sex and age differences were found in the duration of reported sedentary behaviors (see Table 3). Boys spent more time than girls playing computer or video games, whereas girls spent more time than boys using the internet/emailing/electronic media for leisure, doing video calls w/friends or family, sitting while listening to music, sitting talking on the phone/texting, sitting while hanging out with friends/family in person, and doing inactive hobbies. As compared to younger children, older children spent more time playing computer or video games, using the Internet/emailing/ electronic media for leisure, sitting while listening to music, and sitting talking on the phone/texting.

**Table 2:** Descriptive Statistics and Chi-Squares Comparing the Frequencies of Children who Performed Specific Types of Physical Activities on the Previous Day During the Early-COVID-19 period (April–May 2020) by Sex and Age

		Child Sex				Child Age			
	Overall	Male	Female			5–8 yr	9–13 yr		
Types of Physical Activity	*n (%)*	*n (%)*	*n (%)*	*χ*^*2*^	*p*	*n (%)*	*n (%)*	*χ*^*2*^	*p*
Sports practice/training	41 (19.4)	26 (26.0)	15 (13.5)	5.24	.022	26 (22.0)	15 (16.1)	1.16	0.299
Activity classes/lessons	40 (19.0)	13 (13.0)	27 (24.3)	4.39	.036	28 (23.7)	12 (12.9)	3.97	0.053
Free play/unstructured phy. Act.	189 (90.0)	89 (89.9)	100 (90.1)	< 0.01	.963	114 (97.4)	75 (80.6)	16.23	< 0.001
Jogging/running	43 (20.4)	20 (20.0)	23 (20.7)	0.02	.897	25 (21.2)	18 (19.4)	0.11	0.743
Biking	81 (38.4)	35 (35.0)	46 (41.1)	0.92	.337	57 (48.3)	24 (25.8)	11.13	< 0.001
Swimming	18 (8.5)	12 (12.0)	6 (5.4)	2.93	.087	12 (10.2)	6 (6.5)	0.92	0.458
Going for a walk	115 (54.5)	53 (53.0)	62 (55.9)	0.17	.677	67 (56.8)	48 (51.6)	0.56	0.488
Circuit training/conditioning	9 (4.3)	6 (6.0)	3 (2.7)	1.40	.237	2 (1.7)	7 (7.5)	4.33	0.045
Weightlifting	5 (2.4)	3 (3.0)	2 (1.8)	0.33	.568	2 (1.7)	3 (3.2)	0.53	0.656
Other	12 (7.3)	8 (9.6)	4 (4.9)	1.34	.248	3 (3.4)	9 (11.8)	4.28	0.067

N = 211. n (%) = number (proportion) who performed some (vs. none) of the specific type of physical activity

**Table 3:** Descriptive Statistics and t-tests Comparing the Duration (in minutes) of Specific Types of Sedentary Behaviors Performed by Children on the Previous Day During the Early-COVID-19 period (April–May 2020) by Sex and Age

		Child Sex				Child Age Group			
	Overall	Male	Female			5–8 yr	9–13 yr		
Types of Sedentary Behavior	*M (SD)*	*M (SD)*	*M (SD)*	*t*	*p*	*M (SD)*	*M (SD)*	*T*	*p*
Watching television/videos/movies	104.8 (75.0)	95.7 (68.7)	113.0 (79.6)	−1.42	.456	96.7 (70.0)	114.9 (80.0)	−1.23	0.221
Playing computer or video games	50.5 (69.4)	66.3 (79.7)	36.3 (55.2)	2.80	.006	39.0 (60.7)	64.9 (76.8)	−2.78	0.006
Internet/emailing/electronic media for leisure	38.7 (61.9)	30.2 (53.4)	46.4 (68.0)	−2.14	.033	17.5 (38.8)	64.9 (74.2)	−6.62	< 0.001
Doing school-related video calls	39.0 (62.1)	44.4 (65.5)	34.0 (58.7)	1.45	.150	29.3 (49.6)	51.0 (73.4)	−1.89	0.061
Doing video calls w/friends or family	24.5 (37.9)	16.5 (25.3)	31.7 (45.4)	−2.76	.006	20.6 (29.0)	29.3 (46.5)	−0.71	0.481
Doing school-related work	55.1 (69.0)	60.2 (70.4)	50.6 (67.8)	1.43	.153	46.2 (60.7)	66.4 (77.1)	−1.89	0.061
Listening to music	17.2 (40.9)	10.1 (22.5)	23.7 (51.5)	−2.15	.033	7.4 (17.6)	29.5 (56.0)	−3.70	< 0.001
Talking on the phone/texting	13.6 (36.6)	7.8 (24.8)	18.9 (44.1)	−2.66	.008	3.4 (10.3)	26.4 (51.0)	−5.01	< 0.001
Hanging out with friends/family in person	62.2 (60.7)	52.0 (56.0)	71.1 (63.6)	−2.22	.028	64.8 (67.9)	58.8 (50.4)	−0.22	0.823
Reading a book/magazine NOT for school	41.3 (44.1)	34.7 (32.0)	47.2 (52.1)	1.33	.186	41.9 (40.9)	40.6 (47.9)	0.79	0.430
Doing inactive hobbies	40.4 (46.6)	26.0 (29.7)	53.3 (54.5)	−4.43	<.001	42.8 (42.6)	37.3 (50.9)	1.77	0.079
Riding or driving in a car	9.0 (24.2)	8.7 (24.4)	9.2 (24.2)	−0.32	.831	9.4 (25.5)	8.5 (22.7)	0.04	0.969

*n* = 207. Means and standard deviations are presented for raw data. T-tests were conducted on square-root transformed data

Results of the multiple regression analyses for demographic variables predicting square-root transformed total MET-min of PA, total minutes of sitting, minutes of school-related sitting, and minutes of sitting for leisure during the early-COVID-19 period are shown in Table 4. On average, children expended a total of 892.0 (SD = 902.07) MET-min on the previous day through the specific types of physical activities assessed. Children engaged in an average of 91.1 (SD = 109.2) min of sitting for school-related activities, 398.5 (SD = 184.6) min of sitting for leisure activity, and 489.4 (SD = 211.5) min of total sitting on the previous day. After controlling for all the variables in the model, child age was significantly negatively associated with total MET-min of PA. Also, child age was positively associated with minutes of school-related sitting and total minutes of sitting. Lastly, child age and being a girl were positively associated with minutes of sitting for leisure during the early-COVID-19 period after controlling for the child ethnicity (Hispanic vs. non-Hispanic), parent employment status (works full-time vs. does not work full-time), parent marital status (married vs. not married), and annual household income.

**Table 4:** Results of Linear Regression Model for Variables Predicting Children’s Previous-Day Physical Activity and Sedentary Behavior During the Early-COVID-19 period (April–May 2020)

	Total MET-min		Total Sitting min		School Sitting min		Leisure Sitting min	
	*β*	*p*	*β*	*p*	*β*	*p*	*β*	*p*
(Constant)	46.580	< 0.001	14.047	< 0.0001	11.710	0.001	10.035	< 0.0001
Child Age	−0.234	0.001	0.422	< 0.001	0.206	0.004	0.337	< 0.001
Child sex								
Female (vs. Male)	0.010	0.893	0.148	0.027	−0.135	0.062	0.235	0.001
Child ethnicity								
Hispanic (vs. non-Hispanic)	−0.209	0.835	− 0.070	0.287	−0.104	0.142	− 0.024	0.722
Parent Work status								
Full-time (vs. not Full-time)	−0.030	0.682	−0.064	0.375	− 0.189	0.010	0.009	0.898
Parent marital status								
Married (vs. not Married)	−0.233	0.003	0.015	0.838	−0.060	0.440	0.083	0.261
Household Income								
Less than $24,999	−0.051	0.499	0.053	0.449	−0.016	0.838	0.070	0.332
$25,000 - $54,999	0.061	0.405	0.131	0.055	0.002	0.947	0.166	0.018
$55,000 - $94,999	−0.119	0.104	0.177	0.009	0.048	0.514	0.173	0.013
R^2^	0.131	0.001	0.252	< 0.001	0.131	0.001	0.218	< 0.001

*n* = 187. Linear regression analyses were conducted on square-root transformed data. *β =* unstandardized regression coefficient. *R*^*2* =^ percent of variance in the dependent variables that is explained by the combine defect of all the predictor variables in the model. MET-min = Metabolic equivalent of a specific activity x minutes spent doing that activity

### Perceived changes in Children’s physical activity and sedentary behavior

**Fig. 1:**
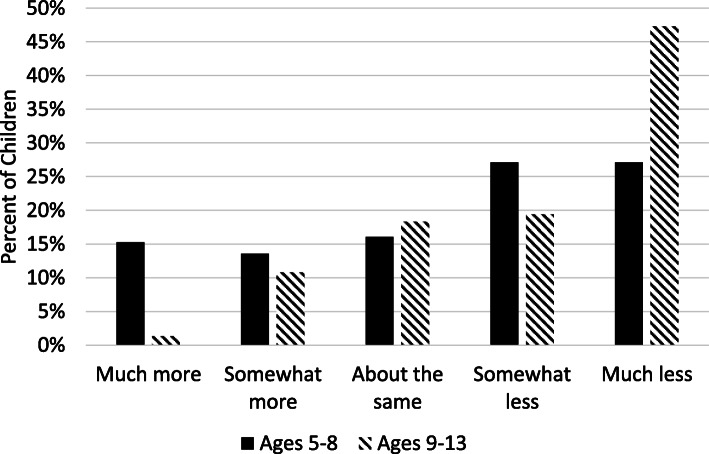
Unadjusted percentages for each category of perceived change in physical activity (from pre-COVID-19 [February 2020] to early-COVID-19 [April–May 2020]) by child age group. *n* = 118 for ages 5–8 and *n* = 93 for ages 9–13

**Fig. 2:**
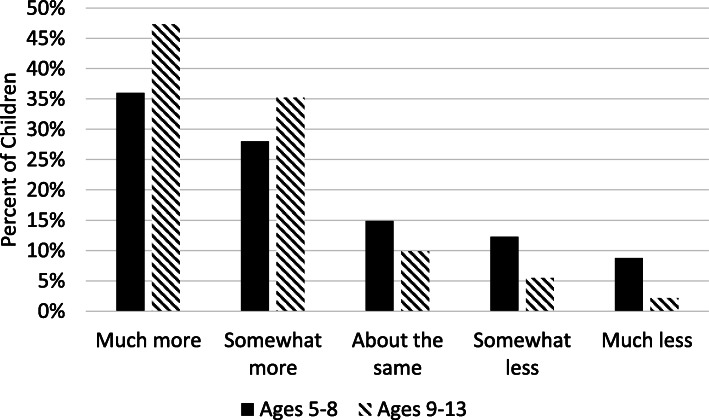
Unadjusted percentages for each category of perceived change in sedentary behavior (from pre-COVID-19 [February 2020] to early-COVID-19 [April–May 2020]) by child age group. *n* = 114 for ages 5–8 and *n* = 91 for ages 9–13

Overall, parents perceived children’s PA had decreased whereas children’s SB had increased between the pre-COVID-19 period (February 2020) and the early-COVID-19 period (April – May 2020). About 36% of parents reported their child had done much less PA in the past 7 days as compared to February 2020, whereas only about 11% of parents reported their child had done much more PA in the past 7 days as compared to February 2020. In contrast, 41% of parents reported their child had done much more sitting in the past 7 days as compared to February 2020, whereas only about 6% of parents reported their child had done much less sitting in the past 7 days as compared to February 2020. Results of the ordinal logistic regression analyses predicting perceived changes in PA and SB from the pre-COVID-19 period to the early-COVID-19 period by child sex and age group controlling for child ethnicity (Hispanic vs. non-Hispanic), annual household income, and parental marital and work status are shown in Table 5. The predictor variable, child age group, was found to contribute to the model for perceived changes in PA (ordered log-odds estimate = −.838, SE = .277, Wald = 9.120, *p* = .003) and the model for perceived changes in SB (ordered log-odds estimate = .623, SE = .280, Wald = 4.947, *p* = .026) controlling for all of the other variables in the model. Parents of older children (ages 9–13) vs. younger children (ages 5–8) were over twice as likely (OR = 2.31, 95% CI [1.34, 3.98]) to have a one-unit change in the perception their children had done less PA in past 7 days as compared to February 2020. The unadjusted proportions of change in each PA category by child age group are shown in Fig. 1. Parents of older children (ages 9–13) vs. younger children (ages 5–8) were half as likely (OR = 0.54, 95% CI [0.31, 0.93]) to have a one-unit change in the perception their children had done less SB in past 7 days as compared to February 2020. The unadjusted proportions of change in each sitting category by child age group are shown in Fig. 2. The odds of perceiving changes in children’s PA and SB were not associated with child sex.

**Table 5:** Results of the Ordinal Logistic Regression Model for Variables Predicting Parents’ Perceived Changes in Children’s Physical Activity and Sedentary Behavior from the pre-COVID-19 period (February 2020) to the Early-COVID-19 period (April–May 2020)

	Perceived Changes in Physical Activity				Perceived Changes in Sedentary Behavior			
	Estimate (SE)	*p*-value	Odds Ratio	OR 95% CI	Estimate (SE)	*p-*value	Odds Ratio	OR 95% CI
Intercept 1 (much more)	−2.697 (1.384)	0.051			−0.210 (1.404)	0.613		
Intercept 2 (somewhat more)	−1.750 (1.375)	0.203			0.658 (1.403)	0.639		
Intercept 3 (about the same)	−0.863 (1.371)	0.529			1.475 (1.407)	0.294		
Intercept 4 (somewhat less)	0.160 (1.369)	0.907			2.530 (1.425)	0.076		
Child age								
9–13 years (vs. 5–8 years)	0.838 (0.277)	0.003	2.312	[1.342, 3.979]	−0.623 (0.280)	0.026	0.536	[0.309, 0.929]
Child sex								
Female (vs. Male)	−0.269 (0.274)	0.327	0.068	[0.447, 1.307]	0.003 (0.279)	0.992	1.003	[0.581, 1.731]
Child ethnicity								
Hispanic (vs. non-Hispanic)	0.867 (0.411)	0.035	2.389	[1.064, 5.323]	−0.364 (0.403)	0.367	0.695	[0.316, 1.531]
Parent work status								
Full-time (vs. not Full-time)	−0.120 (0.294)	0.682	0.887	[0.498, 1.578]	−0.088 (0.299)	0.769	0.916	[0.510, 1.645]
Parent marital status								
Married (vs. not Married)	0.454 (0.449)	0.312	1.575	[0.654, 3.792]	0.640 (0.474)	0.177	1.896	[0.749, 4.802]
Household Income (>$95,000 is ref.)								
< $24,999	−0.658 (0.981)	0.502	0.518	[0.076, 3.540]	1.001 (0.987)	0.316	2.721	[0.385, 19.20]
$25,000 - $54,999	0.796 (0.674)	0.238	2.217	[0.591, 8.306]	−0.715 (0.716)	0.318	0.489	[0.120, 1.992]
$55,000 - $94,999	0.129 (0.367)	0.726	1.138	[0.554, 3.540]	0.340 (0.370)	0.358	1.404	[0.680, 2.901]

*n* = 188 for perceived changes in physical activity, and n = 187 for perceived changes in sedentary behavior. For dependent variable, the reference group is much less. SE = Standard error. *OR* Odds ratio, *CI* Confidence interval

### Changes in locations of physical activity

**Fig. 3:**
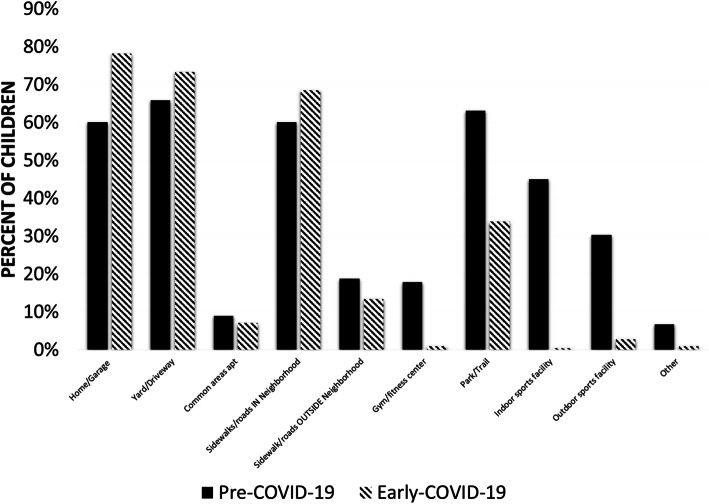
Unadjusted percentages of children whose parents reported that they performed physical activity in each location during the pre-COVID-period (February 20,200 and early-COVID-19 period (April–May 2020). *n* = 187

Generalized estimating equations (GEE) tested changes in the locations of children’s PA from the pre-COVID-19 period (February 2020) to the early-COVID-19 period (April – May 2020). Results found the likelihood of performing PA at home or in the garage (OR = 2.49, 95% CI[1.35, 4.60], Wald = 8.593, *p* = .003) and on sidewalks and roads in their neighborhood (OR = 1.92, 95% CI [1.04,4.60], Wald = 4.28, *p* = .038) increased from the pre-COVID-19 period to the early-COVID-19 period after controlling for child sex, child age group, child ethnicity (Hispanic vs. non-Hispanic), parent employment status (works full-time vs. does not work full-time), parent marital status (married vs. not married), and annual household income. In contrast, the likelihood of performing PA at a park or trail (OR = 0.47, 95% CI [0.23, 0.97], Wald = 4.22, *p* = .040) decreased from the pre-COVID-period to the early-COVID-19 period. The likelihood of children performing PA in their yard or driveway (OR = 1.32, 95% CI [0.76, 2.31], Wald = 0.95, *p* = .329) or on sidewalks and roads outside the neighborhood (OR = 0.76, 95% CI [0.25,2.33], Wald = .288, *p* = .633) did not change. Changes in the locations of children’s PA did not differ by child sex or age group (i.e., interactions between Time × Child sex and Time × Child age group were not significant). GEE models predicting changes in the likelihood of children performing PA in the common areas of their apartments or condos, a gym or fitness center, an indoor sports facility, or an outdoor sports complex did not adequately converge due to low frequencies in certain cells. The unadjusted proportions of children whose parents reported they performed PA in each location during the pre-COVID-period and early-COVID-19 period are shown in Fig. 3.

### Use of remote and streaming Services for Physical Activity

During the early-COVID-19 period, 10.4% of children participated in team sports training sessions or practice through remote or streaming services, 28.9% participated in activity classes or lessons (e.g., martial arts, dance, yoga) through remote or streaming services, and 2.4% participated in remote or streaming classes or sessions provided by a health club or gym. Logistic regression analyses found older children (age 9–13) vs. younger children (ages 5–8) were more than five times as likely to participate in team sports training session or practice through remote or streaming services (OR = 5.40, 95% CI [1.70,17.15], Wald = 8.19, *p* = .004) after controlling for child sex, child ethnicity (Hispanic vs. non-Hispanic), parent employment status (works full-time vs. does not work full-time), parent marital status (married vs. not married), and annual household income. There were no other differences in the likelihood of participating in team sports, activity classes or lesson, or sessions provided by a health club or gym by child sex or age group.

## Discussion

This project is one of the first known studies to examine the early effects of the COVID-19 pandemic on PA and SB among U.S. children. Data were collected during a period of time (April – May 2020) when the most restrictive policies were in place to prevent the spread of the virus, including the closure of primary and secondary schools in all 50 states, the cancellation of team sports and activity classes for youth, and the closure of public parks and playgrounds. Generally, parents perceived children’s PA had decreased whereas children’s SB had increased between the pre-COVID-19 period and the early-COVID-19 period. The locations of children’s PA also changed drastically, with more children performing PA at home or in the garage, and on sidewalks and roads in their neighborhood during the early-COVID-19 period. Overall, COVID-19 restrictions seemed to have a larger impact on the PA and SB of older children and girls. Of public health concern is these short-term changes in behavior in reaction to COVID-19 may become permanently entrenched, leading to increased risk of obesity, diabetes, and cardiovascular disease in children as they get older. Therefore, programmatic and policy strategies should be geared towards promoting PA and reducing SB during the next few months of the pandemic.

Results found children had different patterns of activity than what was seen before COVID-19. The most frequently reported physical activities during the early-COVID-19 period were free play/unstructured PA (e.g., running around, tag, other active games) and going for a walk. This pattern was not surprising given school closures and team sports/activity class cancellations, most children were spending their entire days at home with little access to structured activities. However, it offers a contrast to typical patterns of children’s PA, suggesting that unstructured and free play activities have become less common as children’s time has been increasingly consumed by organized activities [31]. Younger children (ages 5–8) were more likely to participate in free play/unstructured activities as, well as biking and scootering/skateboarding/roller skating than older children (ages 9–13), most likely reflecting developmental preferences for these types of activities [32]. Given the potential lack of access to organized sports and activity classes over the next 6–12 months due to concerns of COVID-19 spreading in those settings, efforts to promote free and unstructured PA among older children should be made perhaps by playing with younger siblings or through socially-distant or masked play dates with non-household peers.

Of the SB assessed, children spent the most time watching television/videos/movies, sitting while hanging out with friends or family in person, doing school-related work, and playing computer or video games during the early-COVID-19 period. Interestingly, school-related sedentary time, including school-related video calls and doing school-related work, only accounted for about 90 min on the day assessed (which included weekdays and weekend days). In contrast, sitting for leisure activities (e.g., video games, TV, internet, hanging out with family) accounted for over 8 h on the day that was assessed. It should be noted that the item asking about “sitting while hanging out with family or friends in person” would ideally be assessed through two separate questions because it is recommended that individuals limit in-person contact with people who live outside their household during the COVID-19 pandemic. Overall, these data suggest during the early-COVID-19 period, children overwhelmingly spent their unstructured free time doing sedentary pursuits instead of physical activities. Girls and older children generally spent more time in these sedentary behaviors than boys and younger children, suggesting that sex and age differences that are typically observed in sitting time [33, 34] may be exacerbated during the COVID-19 pandemic, placing girls and older children at even greater risk of health concerns due to physical inactivity such as obesity and metabolic dysregulation.

Patterns observed in parents’ perceptions of changes in children’s PA and SB between the pre- and early-COVID-19 periods further underscore the heightened risk of the pandemic for older children. While parents generally perceived their children’s PA had decreased and their SB had increased, these changes were much more pronounced for older children (ages 9–13). Twice as many parents of older (vs. younger) children reported their child had done much less PA in the past 7 days as compared to the pre-COVID-19 period. Although PA typically declines and SB increases as children get older [33, 35–38], the COVID-19 pandemic may be accelerating these development changes. Of great concern is older children may adopt new behavioral habits of physical inactivity during the pandemic that are extremely difficult to change when pandemic-related school closure and organized sports cancellations end. Although objective monitor-based activity data are needed to lend additional validity to these observed effects of the pandemic on older children’s activity levels, preventive actions may need to be taken before these types of data can become available. If intermittent school closures and interruptions continue through the 2020–2021 academic year, upper elementary and middle schools may make concerted efforts to incorporate physical activities into distance-learning curriculum such as activity breaks [39], physically active subject-based lessons [40], or online physical education. Gaps could also be addressed through the development and delivery of free online physical activities and lessons through non-profit and government entities.

Not surprisingly, the locations of children’s PA changed drastically between the pre- and early-COVID-19 periods, which may explain the perceived declines in children’s PA reported by parents. The significant increase in the proportion of children who performed PA at home or in the garage most likely reflects common pre-COVID-19 locations for PA such as indoor and outdoor sports facilities, and parks were not available. It was somewhat unexpected, however, to see that more of children’s PA appeared to be displaced to inside the home or garage than to the yard or driveway. Barriers to outdoor PA in one’s yard or driveway such as inclement weather [41, 42] (especially in the Northern states in late April) or lack of outdoor space available at one’s place of residence [43] may explain this pattern. The significant increase in children’s PA occurring on the sidewalks and roads in their immediate neighborhood represents a unique trend observed during the COVID-19 pandemic. More people staying at home results in lower traffic volume on city streets [9] and increased space for children’s PA. In some cases, cities even placed signage on local streets to slow down traffic, so pedestrians and bicyclists could use the street to allow for social-distancing [44, 45]. Children’s increased usage of local streets and sidewalks for PA parallels the substantial percentage of parents who reported their child spent time going for a walk on the previous day. Overall, these trends point to a promising opportunity for city planners and local governments who are searching for ways to promote PA during the COVID-19 pandemic.

One of the most unique findings to come out of this study was the substantial percentage of children who had begun using remote and streaming services to engage in PA during the early-COVID-19 period. Prior to the pandemic, using online technologies to deliver sports training and activity classes had been growing among adults [46], but had been slow to be adopted among children [47]. In the current study, over 10 % of children had participated in team sports training sessions or practice and almost a third of children had participated in activity classes or lessons (e.g., martial arts, dance, yoga) through remote or streaming services, representing a significant departure from how children were typically accessing organized PA prior to the pandemic. To stay in business, many youth sport, martial arts, and dance professionals shifted to online delivery during the early-COVID-19 period [48]. In the future, children may be receptive of these technologies as a replacement for in-person forms of organized PA, which can be time- and resource-consuming for families in terms of transportation and other costs. Whether the use of remote and streaming services to promote children’s PA is an effective medium to teach skills, can be offered equitably across diverse socioeconomic groups, and continue after the pandemic needs to be better investigated.

Methodological strengths of the study included the timeliness of the baseline survey during the early-COVID-19 period and wide geographic representation. Yet, some limitations existed. Asking parents to report on children’s PA and SB was necessary for the youngest children in the sample (e.g., ages 5–7) who may not yet have the reading or cognitive capabilities to reliably report for themselves. However, parents may be less aware of the amount of time their middle-school children (e.g., ages 11–13) are spending on activities even when both children and parents may be at home together due to the pandemic. Along these same lines, asking parents to compare their children’s early-COVID-19 (April–May 2020) PA and SB levels to their pre-COVID-19 (February 2020) levels may introduce some reporting error and biases because most school-aged children were not in their parents’ presence during school hours in February 2020. Also, some parents with essential jobs working out of the home and parents who were working full-time at home during the early-COVID-19 period may not have an accurate representation of how much time their children were spending in each type of activity. Furthermore, parents’ levels of physical activity were not assessed, so it cannot be determined how children may be role-modeling their parents. Lastly, the survey respondents were mainly more highly educated mothers with higher household income levels. Findings may not extend to children whose parents have not attained a college degree or who reside in lower income households. It will be useful for future research on the impact of COVID-19 on children’s PA and SB to collect data from a more racially-ethnically diverse sample and among lower income families. Our sample was not representative compared to U.S. demographic data. In 2018, 25% of U.S. children identified as Hispanic while 50% identified as non-Hispanic White [49] whereas our sample 13% Hispanic and 77% non-Hispanic White. The sample was not geographically equally distributed almost a third of participants resided in California. Compared to U.S. data [50], our sample had fewer adults employed full-time (61%); however, employment changes due to COVID-19 have not been accounted for in U.S. demographic data reports.

## Conclusions

Overall, results from this study suggest U.S. children performed less PA and engaged in more SB during the early-COVID-19 period as compared to before the pandemic. Although school and park closures and cancellations of team sports and organized activity classes were necessary steps to mitigate the spread of the virus and allow healthcare facilities to build capacity, they appear to have had a profound impact on children’s PA and SB levels—especially among older children (ages 9–13). In order to avoid permanent changes in behavior extending beyond the duration of the COVID-19 closures, measures must be taken over the summer and fall of 2020 to promote home- and neighborhood-based PA during children’s leisure time.

## Data Availability

The datasets used and/or analyzed during the current study are available from the corresponding author on reasonable request.

## Abbreviations

PA: Physical activity
SB: Sedentary behavior
U.S.: United States of America
MET: Metabolic equivalent
OR: Odds ratio
CI: Confidence interval
SE: Standard error
